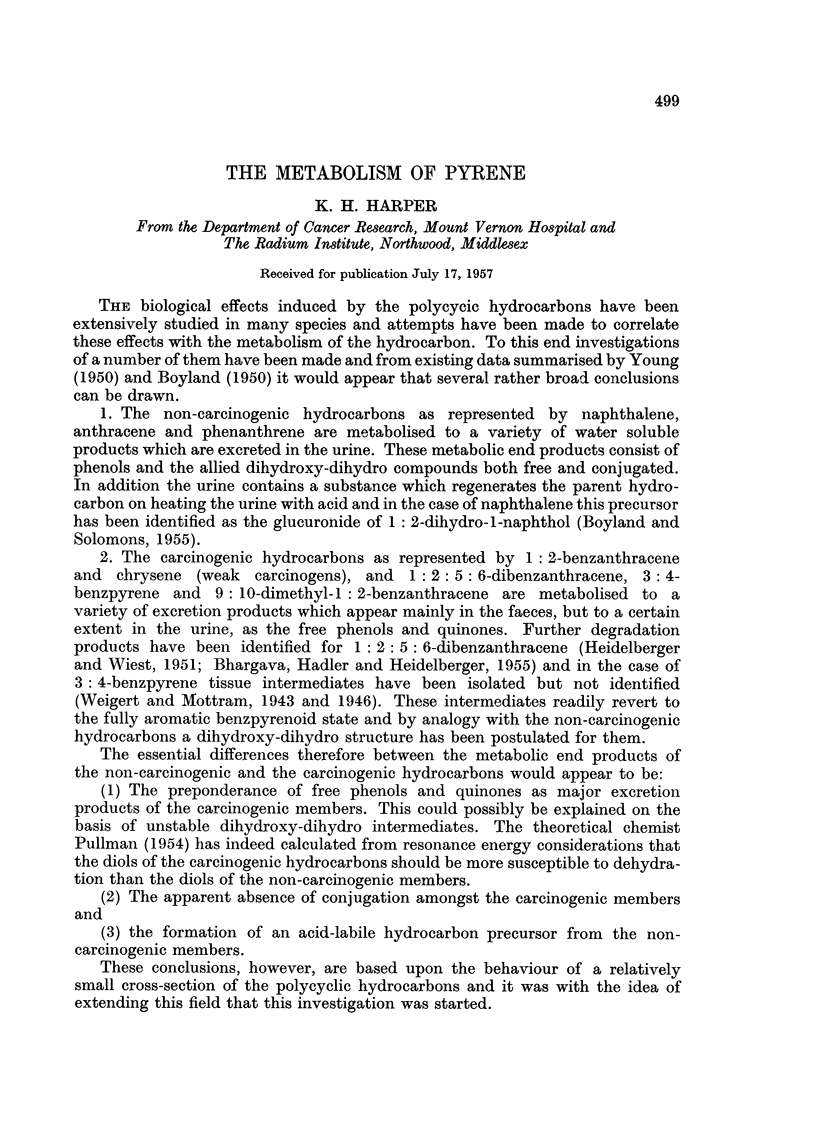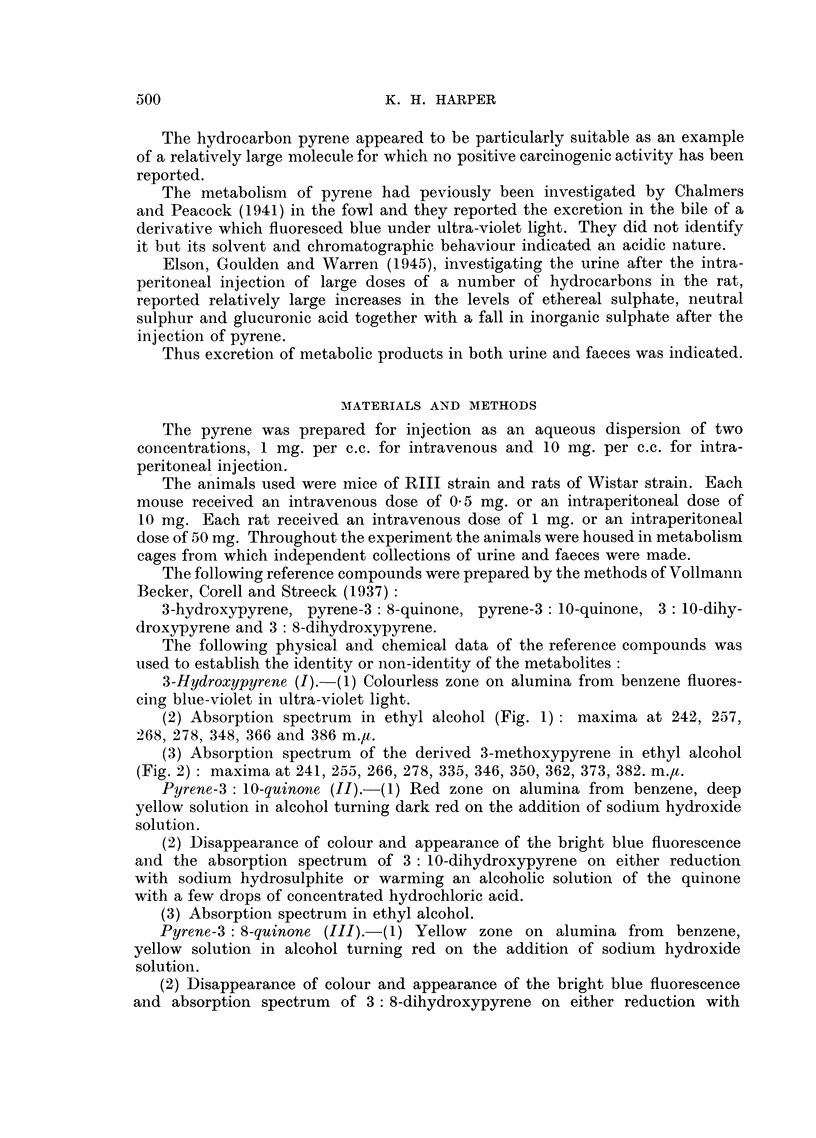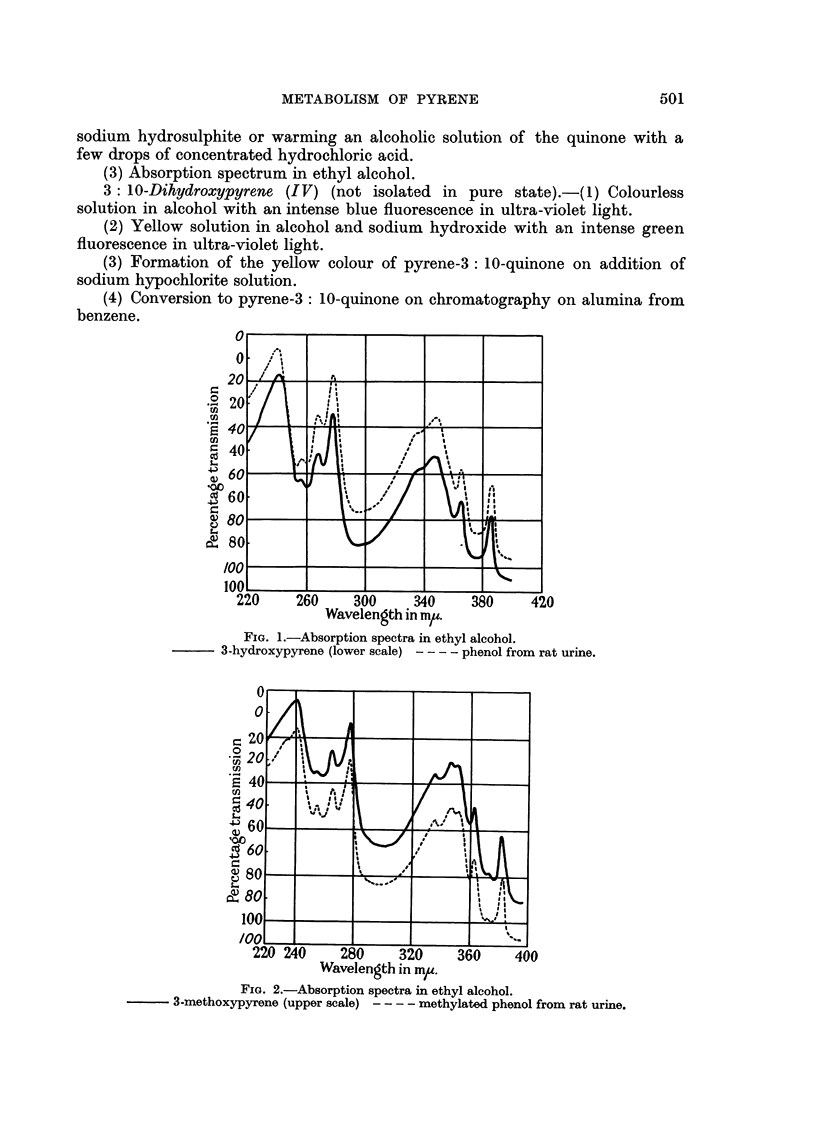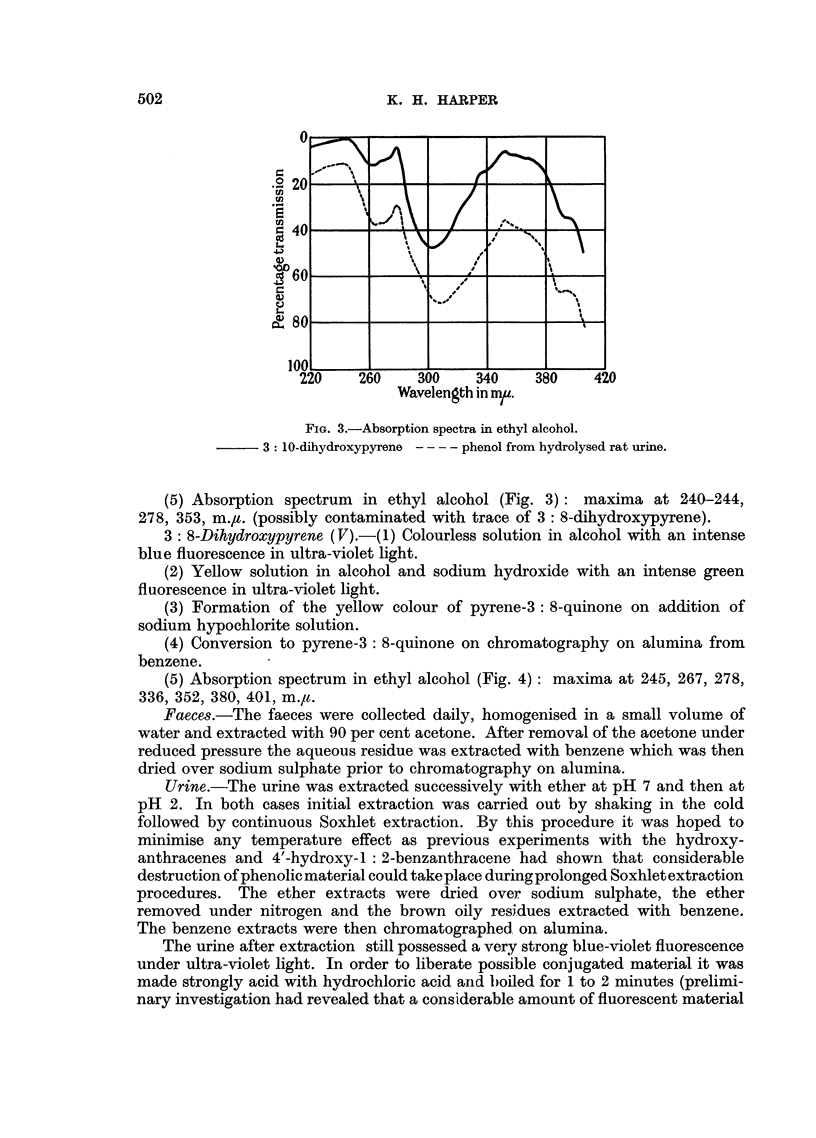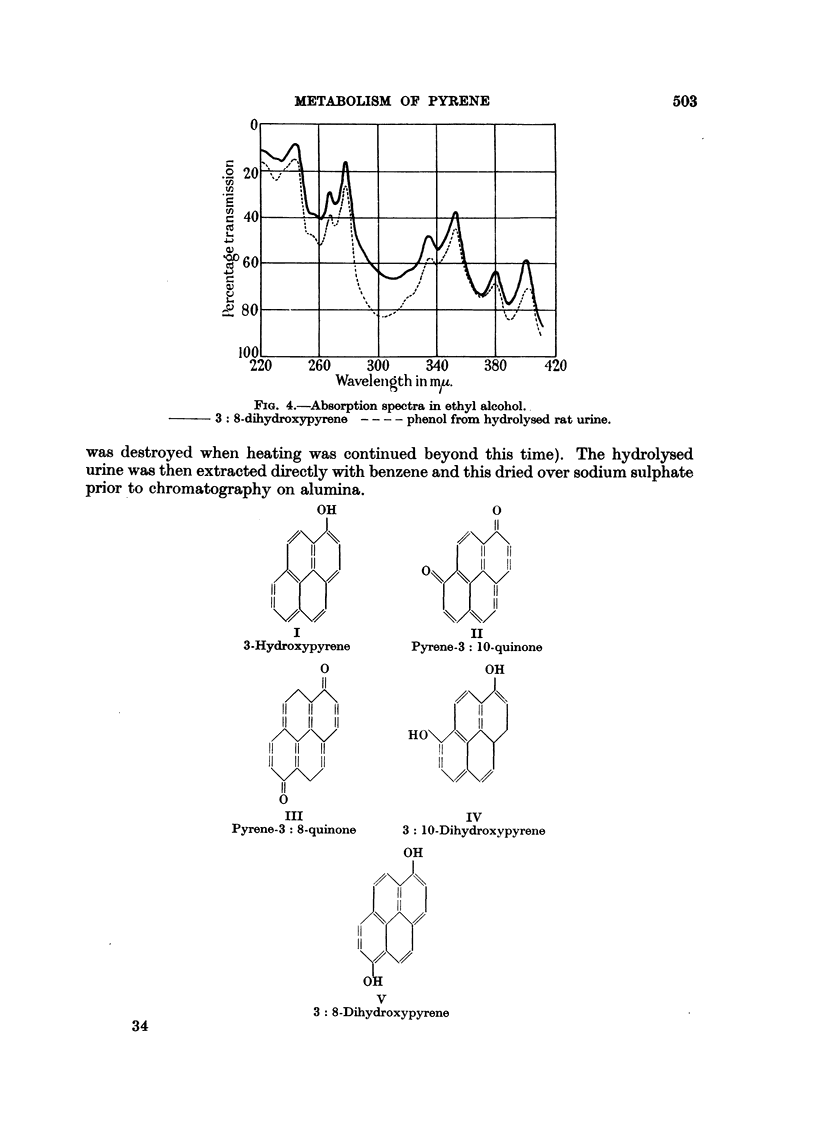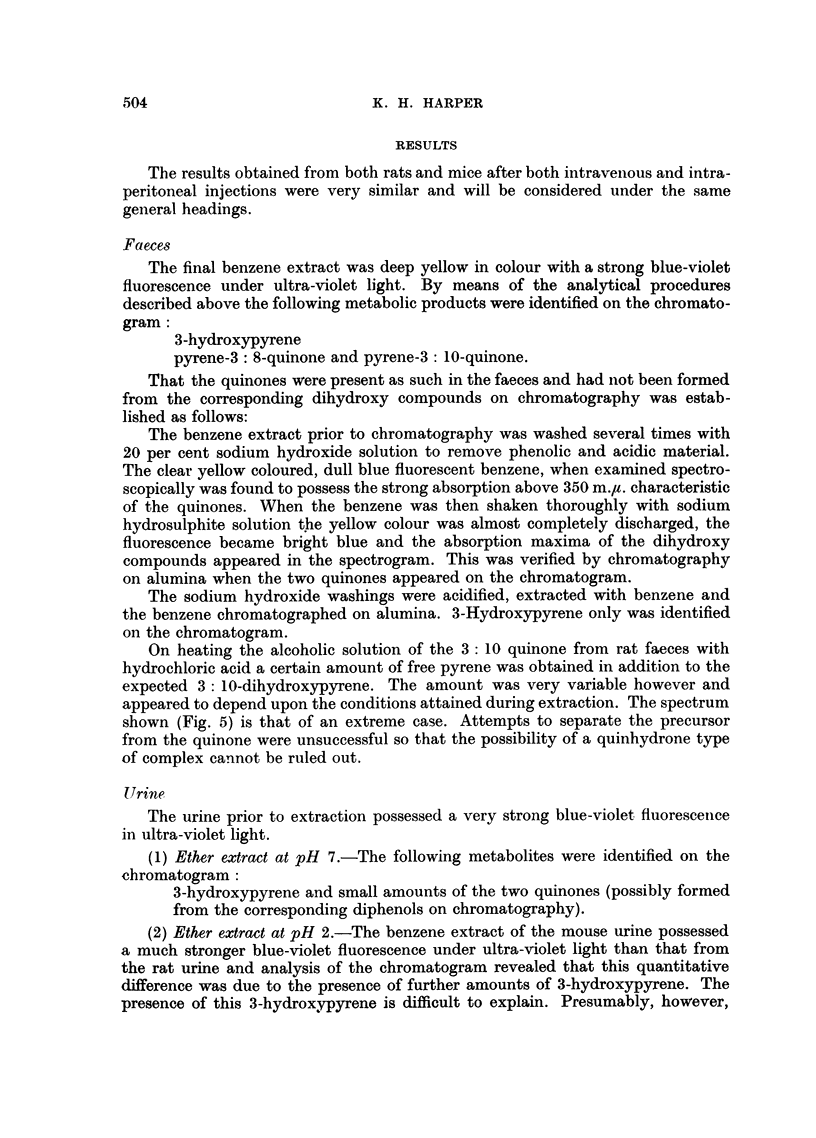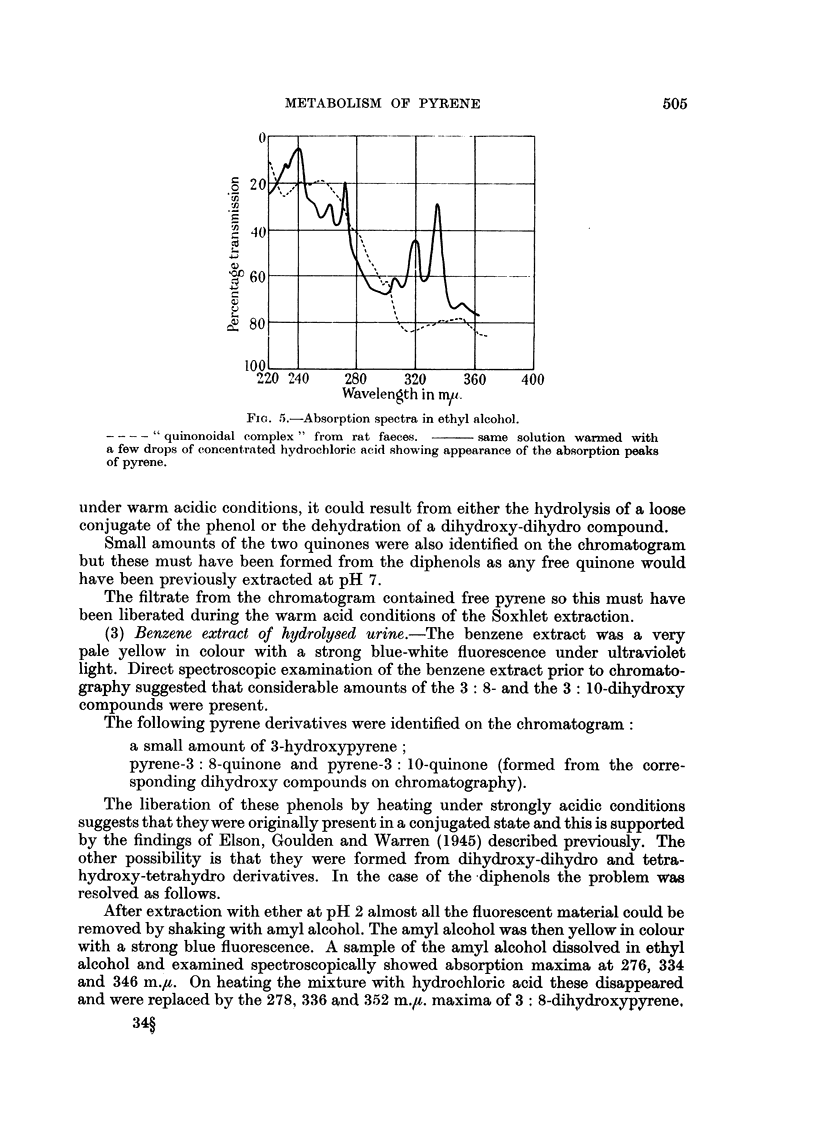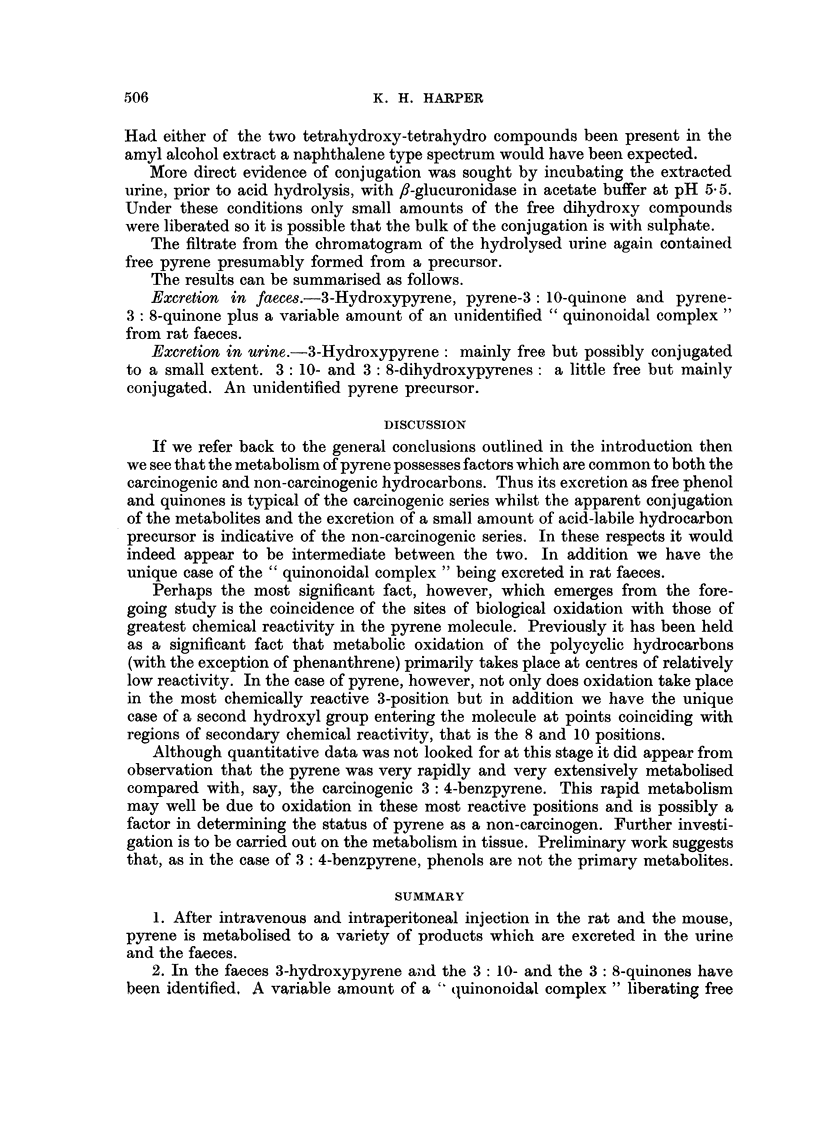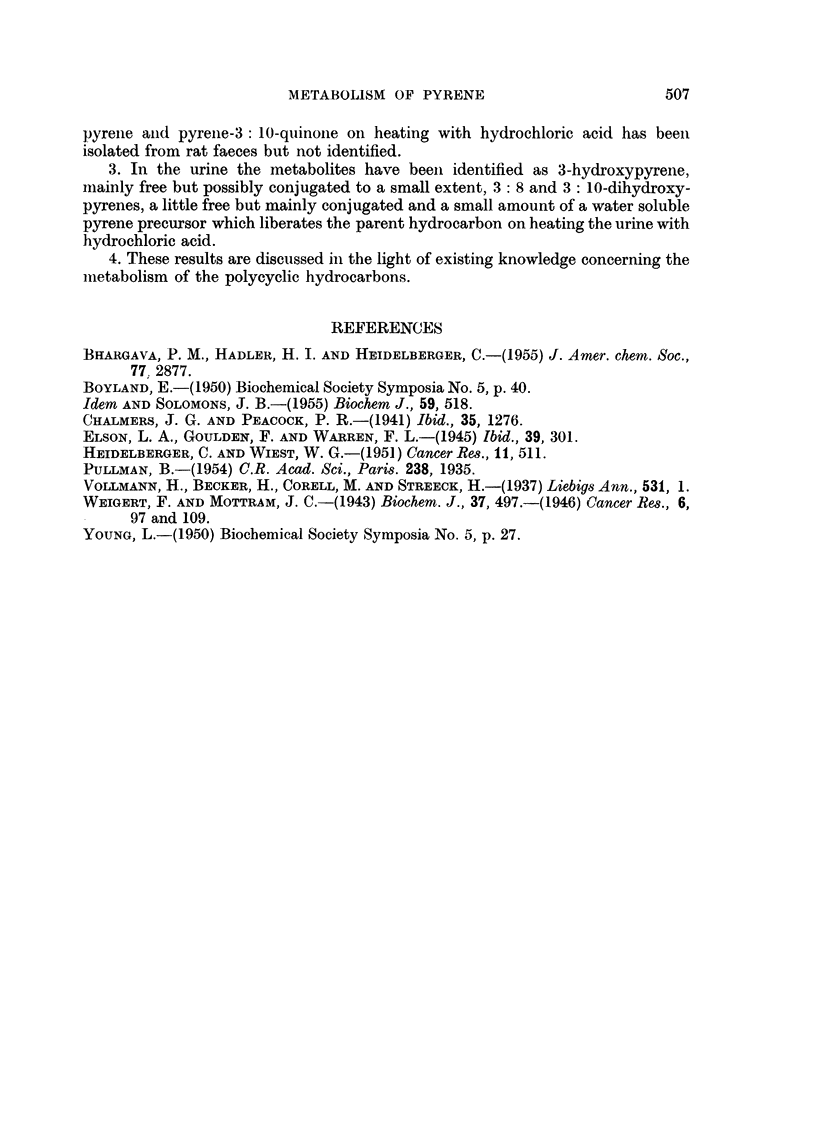# The Metabolism of Pyrene

**DOI:** 10.1038/bjc.1957.61

**Published:** 1957-09

**Authors:** K. H. Harper


### THE METABOLISM OF PYRENE

K. H. HARPER

*From the Department of Cancer Research, Mount Vernon Hospital and The Radium Institute, Northwood, Middlesex*

Received for publication July 17, 1957

THE biological effects induced by the polycycic hydrocarbons have been extensively studied in many species and attempts have been made to correlate these effects with the metabolism of the hydrocarbon. To this end investigations of a number of them have been made and from existing data summarised by Young (1950) and Boyland (1950) it would appear that several rather broad conclusions can be drawn.

1. The non-carcinogenic hydrocarbons as represented by naphthalene, anthracene and phenanthrene are metabolised to a variety of water soluble products which are excreted in the urine. These metabolic end products consist of phenols and the allied dihydroxy-dihydro compounds both free and conjugated. In addition the urine contains a substance which regenerates the parent hydrocarbon on heating the urine with acid and in the case of naphthalene this precursor has been identified as the glucuronide of 1 : 2-dihydro-1-naphthol (Boyland and Solomons, 1955).

2. The carcinogenic hydrocarbons as represented by 1 : 2-benzanthracene and chrysene (weak carcinogens), and 1 : 2 : 5 : 6-dibenzanthracene, 3 : 4-benzpyrene and 9 : 10-dimethyl-1 : 2-benzanthracene are metabolised to a variety of excretion products which appear mainly in the faeces, but to a certain extent in the urine, as the free phenols and quinones. Further degradation products have been identified for 1 : 2 : 5 : 6-dibenzanthracene (Heidelberger and Wiest, 1951; Bhargava, Hadler and Heidelberger, 1955) and in the case of 3 : 4-benzpyrene tissue intermediates have been isolated but not identified (Weigert and Mottram, 1943 and 1946). These intermediates readily revert to the fully aromatic benzpyrenoid state and by analogy with the non-carcinogenic hydrocarbons a dihydroxy-dihydro structure has been postulated for them.

The essential differences therefore between the metabolic end products of the non-carcinogenic and the carcinogenic hydrocarbons would appear to be:

(1) The preponderance of free phenols and quinones as major excretion products of the carcinogenic members. This could possibly be explained on the basis of unstable dihydroxy-dihydro intermediates. The theoretical chemist Pullman (1954) has indeed calculated from resonance energy considerations that the diols of the carcinogenic hydrocarbons should be more susceptible to dehydration than the diols of the non-carcinogenic members.

(2) The apparent absence of conjugation amongst the carcinogenic members and

(3) the formation of an acid-labile hydrocarbon precursor from the non-carcinogenic members.

These conclusions, however, are based upon the behaviour of a relatively small cross-section of the polycyclic hydrocarbons and it was with the idea of extending this field that this investigation was started.

The hydrocarbon pyrene appeared to be particularly suitable as an example of a relatively large molecule for which no positive carcinogenic activity has been reported.

The metabolism of pyrene had peviously been investigated by Chalmers and Peacock (1941) in the fowl and they reported the excretion in the bile of a derivative which fluoresced blue under ultra-violet light. They did not identify it but its solvent and chromatographic behaviour indicated an acidic nature.

Elson, Goulden and Warren (1945), investigating the urine after the intraperitoneal injection of large doses of a number of hydrocarbons in the rat, reported relatively large increases in the levels of ethereal sulphate, neutral sulphur and glucuronic acid together with a fall in inorganic sulphate after the injection of pyrene.

Thus excretion of metabolic products in both urine and faeces was indicated.

### MATERIALS AND METHODS

The pyrene was prepared for injection as an aqueous dispersion of two concentrations, 1 mg. per c.c. for intravenous and 10 mg. per c.c. for intraperitoneal injection.

The animals used were mice of RIII strain and rats of Wistar strain. Each mouse received an intravenous dose of 0·5 mg. or an intraperitoneal dose of 10 mg. Each rat received an intravenous dose of 1 mg. or an intraperitoneal dose of 50 mg. Throughout the experiment the animals were housed in metabolism cages from which independent collections of urine and faeces were made.

The following reference compounds were prepared by the methods of Vollmann Becker, Corell and Streeck (1937) :

3-hydroxypyrene, pyrene-3 : 8-quinone, pyrene-3 : 10-quinone, 3 : 10-dihydroxypyrene and 3 : 8-dihydroxypyrene.

The following physical and chemical data of the reference compounds was used to establish the identity or non-identity of the metabolites :

3-*Hydroxypyrene* (*I*).—(1) Colourless zone on alumina from benzene fluorescing blue-violet in ultra-violet light.

(2) Absorption spectrum in ethyl alcohol (Fig. 1) : maxima at 242, 257, 268, 278, 348, 366 and 386 m.$\mu$.

(3) Absorption spectrum of the derived 3-methoxypyrene in ethyl alcohol (Fig. 2) : maxima at 241, 255, 266, 278, 335, 346, 350, 362, 373, 382. m.$\mu$.

*Pyrene*-3 : 10-*quinone* (*II*).—(1) Red zone on alumina from benzene, deep yellow solution in alcohol turning dark red on the addition of sodium hydroxide solution.

(2) Disappearance of colour and appearance of the bright blue fluorescence and the absorption spectrum of 3 : 10-dihydroxypyrene on either reduction with sodium hydrosulphite or warming an alcoholic solution of the quinone with a few drops of concentrated hydrochloric acid.

(3) Absorption spectrum in ethyl alcohol.

*Pyrene*-3 : 8-*quinone* (*III*).—(1) Yellow zone on alumina from benzene, yellow solution in alcohol turning red on the addition of sodium hydroxide solution.

(2) Disappearance of colour and appearance of the bright blue fluorescence and absorption spectrum of 3 : 8-dihydroxypyrene on either reduction with sodium hydrosulphite or warming an alcoholic solution of the quinone with a few drops of concentrated hydrochloric acid.

(3) Absorption spectrum in ethyl alcohol.

3 : 10-*Dihydroxypyrene* (*IV*) (not isolated in pure state).—(1) Colourless solution in alcohol with an intense blue fluorescence in ultra-violet light.

(2) Yellow solution in alcohol and sodium hydroxide with an intense green fluorescence in ultra-violet light.

(3) Formation of the yellow colour of pyrene-3 : 10-quinone on addition of sodium hypochlorite solution.

(4) Conversion to pyrene-3 : 10-quinone on chromatography on alumina from benzene.

Fig. 1.—Absorption spectra in ethyl alcohol.
——— 3-hydroxypyrene (lower scale) – – – – phenol from rat urine.

Fig. 2.—Absorption spectra in ethyl alcohol.
——— 3-methoxypyrene (upper scale) – – – – methylated phenol from rat urine.

FIG. 3.—Absorption spectra in ethyl alcohol.
——— 3 : 10-dihydroxypyrene – – – – phenol from hydrolysed rat urine.

(5) Absorption spectrum in ethyl alcohol (Fig. 3): maxima at 240–244, 278, 353, m.μ. (possibly contaminated with trace of 3 : 8-dihydroxypyrene).

3 : 8-*Dihydroxypyrene* (*V*).—(1) Colourless solution in alcohol with an intense blue fluorescence in ultra-violet light.

(2) Yellow solution in alcohol and sodium hydroxide with an intense green fluorescence in ultra-violet light.

(3) Formation of the yellow colour of pyrene-3 : 8-quinone on addition of sodium hypochlorite solution.

(4) Conversion to pyrene-3 : 8-quinone on chromatography on alumina from benzene.

(5) Absorption spectrum in ethyl alcohol (Fig. 4): maxima at 245, 267, 278, 336, 352, 380, 401, m.μ.

*Faeces*.—The faeces were collected daily, homogenised in a small volume of water and extracted with 90 per cent acetone. After removal of the acetone under reduced pressure the aqueous residue was extracted with benzene which was then dried over sodium sulphate prior to chromatography on alumina.

*Urine*.—The urine was extracted successively with ether at pH 7 and then at pH 2. In both cases initial extraction was carried out by shaking in the cold followed by continuous Soxhlet extraction. By this procedure it was hoped to minimise any temperature effect as previous experiments with the hydroxy-anthracenes and 4′-hydroxy-1 : 2-benzanthracene had shown that considerable destruction of phenolic material could take place during prolonged Soxhlet extraction procedures. The ether extracts were dried over sodium sulphate, the ether removed under nitrogen and the brown oily residues extracted with benzene. The benzene extracts were then chromatographed on alumina.

The urine after extraction still possessed a very strong blue-violet fluorescence under ultra-violet light. In order to liberate possible conjugated material it was made strongly acid with hydrochloric acid and boiled for 1 to 2 minutes (preliminary investigation had revealed that a considerable amount of fluorescent material

FIG. 4.—Absorption spectra in ethyl alcohol.
——— 3 : 8-dihydroxypyrene – – – – phenol from hydrolysed rat urine.

was destroyed when heating was continued beyond this time). The hydrolysed urine was then extracted directly with benzene and this dried over sodium sulphate prior to chromatography on alumina.

I
3-Hydroxypyrene

II
Pyrene-3 : 10-quinone

III
Pyrene-3 : 8-quinone

IV
3 : 10-Dihydroxypyrene

V
3 : 8-Dihydroxypyrene

### RESULTS

The results obtained from both rats and mice after both intravenous and intraperitoneal injections were very similar and will be considered under the same general headings.

### *Faeces*

The final benzene extract was deep yellow in colour with a strong blue-violet fluorescence under ultra-violet light. By means of the analytical procedures described above the following metabolic products were identified on the chromatogram :

3-hydroxypyrene
pyrene-3 : 8-quinone and pyrene-3 : 10-quinone.

That the quinones were present as such in the faeces and had not been formed from the corresponding dihydroxy compounds on chromatography was established as follows:

The benzene extract prior to chromatography was washed several times with 20 per cent sodium hydroxide solution to remove phenolic and acidic material. The clear yellow coloured, dull blue fluorescent benzene, when examined spectroscopically was found to possess the strong absorption above 350 m.$\mu$. characteristic of the quinones. When the benzene was then shaken thoroughly with sodium hydrosulphite solution the yellow colour was almost completely discharged, the fluorescence became bright blue and the absorption maxima of the dihydroxy compounds appeared in the spectrogram. This was verified by chromatography on alumina when the two quinones appeared on the chromatogram.

The sodium hydroxide washings were acidified, extracted with benzene and the benzene chromatographed on alumina. 3-Hydroxypyrene only was identified on the chromatogram.

On heating the alcoholic solution of the 3 : 10 quinone from rat faeces with hydrochloric acid a certain amount of free pyrene was obtained in addition to the expected 3 : 10-dihydroxypyrene. The amount was very variable however and appeared to depend upon the conditions attained during extraction. The spectrum shown (Fig. 5) is that of an extreme case. Attempts to separate the precursor from the quinone were unsuccessful so that the possibility of a quinhydrone type of complex cannot be ruled out.

### *Urine*

The urine prior to extraction possessed a very strong blue-violet fluorescence in ultra-violet light.

(1) *Ether extract at pH* 7.—The following metabolites were identified on the chromatogram :

3-hydroxypyrene and small amounts of the two quinones (possibly formed from the corresponding diphenols on chromatography).

(2) *Ether extract at pH* 2.—The benzene extract of the mouse urine possessed a much stronger blue-violet fluorescence under ultra-violet light than that from the rat urine and analysis of the chromatogram revealed that this quantitative difference was due to the presence of further amounts of 3-hydroxypyrene. The presence of this 3-hydroxypyrene is difficult to explain. Presumably, however,

FIG. 5.—Absorption spectra in ethyl alcohol.

– – – – " quinonoidal complex " from rat faeces. ——— same solution warmed with a few drops of concentrated hydrochloric acid showing appearance of the absorption peaks of pyrene.

under warm acidic conditions, it could result from either the hydrolysis of a loose conjugate of the phenol or the dehydration of a dihydroxy-dihydro compound.

Small amounts of the two quinones were also identified on the chromatogram but these must have been formed from the diphenols as any free quinone would have been previously extracted at pH 7.

The filtrate from the chromatogram contained free pyrene so this must have been liberated during the warm acid conditions of the Soxhlet extraction.

(3) *Benzene extract of hydrolysed urine.*—The benzene extract was a very pale yellow in colour with a strong blue-white fluorescence under ultraviolet light. Direct spectroscopic examination of the benzene extract prior to chromatography suggested that considerable amounts of the 3 : 8- and the 3 : 10-dihydroxy compounds were present.

The following pyrene derivatives were identified on the chromatogram :

a small amount of 3-hydroxypyrene ; pyrene-3 : 8-quinone and pyrene-3 : 10-quinone (formed from the corresponding dihydroxy compounds on chromatography).

The liberation of these phenols by heating under strongly acidic conditions suggests that they were originally present in a conjugated state and this is supported by the findings of Elson, Goulden and Warren (1945) described previously. The other possibility is that they were formed from dihydroxy-dihydro and tetrahydroxy-tetrahydro derivatives. In the case of the diphenols the problem was resolved as follows.

After extraction with ether at pH 2 almost all the fluorescent material could be removed by shaking with amyl alcohol. The amyl alcohol was then yellow in colour with a strong blue fluorescence. A sample of the amyl alcohol dissolved in ethyl alcohol and examined spectroscopically showed absorption maxima at 276, 334 and 346 m.μ. On heating the mixture with hydrochloric acid these disappeared and were replaced by the 278, 336 and 352 m.μ. maxima of 3 : 8-dihydroxypyrene.

Had either of the two tetrahydroxy-tetrahydro compounds been present in the amyl alcohol extract a naphthalene type spectrum would have been expected.

More direct evidence of conjugation was sought by incubating the extracted urine, prior to acid hydrolysis, with $\beta$-glucuronidase in acetate buffer at pH 5·5. Under these conditions only small amounts of the free dihydroxy compounds were liberated so it is possible that the bulk of the conjugation is with sulphate.

The filtrate from the chromatogram of the hydrolysed urine again contained free pyrene presumably formed from a precursor.

The results can be summarised as follows.

*Excretion in faeces.*—3-Hydroxypyrene, pyrene-3 : 10-quinone and pyrene-3 : 8-quinone plus a variable amount of an unidentified " quinonoidal complex " from rat faeces.

*Excretion in urine.*—3-Hydroxypyrene : mainly free but possibly conjugated to a small extent. 3 : 10- and 3 : 8-dihydroxypyrenes : a little free but mainly conjugated. An unidentified pyrene precursor.

### DISCUSSION

If we refer back to the general conclusions outlined in the introduction then we see that the metabolism of pyrene possesses factors which are common to both the carcinogenic and non-carcinogenic hydrocarbons. Thus its excretion as free phenol and quinones is typical of the carcinogenic series whilst the apparent conjugation of the metabolites and the excretion of a small amount of acid-labile hydrocarbon precursor is indicative of the non-carcinogenic series. In these respects it would indeed appear to be intermediate between the two. In addition we have the unique case of the " quinonoidal complex " being excreted in rat faeces.

Perhaps the most significant fact, however, which emerges from the foregoing study is the coincidence of the sites of biological oxidation with those of greatest chemical reactivity in the pyrene molecule. Previously it has been held as a significant fact that metabolic oxidation of the polycyclic hydrocarbons (with the exception of phenanthrene) primarily takes place at centres of relatively low reactivity. In the case of pyrene, however, not only does oxidation take place in the most chemically reactive 3-position but in addition we have the unique case of a second hydroxyl group entering the molecule at points coinciding with regions of secondary chemical reactivity, that is the 8 and 10 positions.

Although quantitative data was not looked for at this stage it did appear from observation that the pyrene was very rapidly and very extensively metabolised compared with, say, the carcinogenic 3 : 4-benzpyrene. This rapid metabolism may well be due to oxidation in these most reactive positions and is possibly a factor in determining the status of pyrene as a non-carcinogen. Further investigation is to be carried out on the metabolism in tissue. Preliminary work suggests that, as in the case of 3 : 4-benzpyrene, phenols are not the primary metabolites.

### SUMMARY

1. After intravenous and intraperitoneal injection in the rat and the mouse, pyrene is metabolised to a variety of products which are excreted in the urine and the faeces.

2. In the faeces 3-hydroxypyrene and the 3 : 10- and the 3 : 8-quinones have been identified. A variable amount of a " quinonoidal complex " liberating free pyrene and pyrene-3 : 10-quinone on heating with hydrochloric acid has been isolated from rat faeces but not identified.

3. In the urine the metabolites have been identified as 3-hydroxypyrene, mainly free but possibly conjugated to a small extent, 3 : 8 and 3 : 10-dihydroxypyrenes, a little free but mainly conjugated and a small amount of a water soluble pyrene precursor which liberates the parent hydrocarbon on heating the urine with hydrochloric acid.

4. These results are discussed in the light of existing knowledge concerning the metabolism of the polycyclic hydrocarbons.

### REFERENCES

BHARGAVA, P. M., HADLER, H. I. AND HEIDELBERGER, C.—(1955) *J. Amer. chem. Soc.*, **77**, 2877.

BOYLAND, E.—(1950) Biochemical Society Symposia No. 5, p. 40.

*Idem* AND SOLOMONS, J. B.—(1955) *Biochem J.*, **59**, 518.

CHALMERS, J. G. AND PEACOCK, P. R.—(1941) *Ibid.*, **35**, 1276.

ELSON, L. A., GOULDEN, F. AND WARREN, F. L.—(1945) *Ibid.*, **39**, 301.

HEIDELBERGER, C. AND WIEST, W. G.—(1951) *Cancer Res.*, **11**, 511.

PULLMAN, B.—(1954) *C.R. Acad. Sci., Paris.* **238**, 1935.

VOLLMANN, H., BECKER, H., CORELL, M. AND STREECK, H.—(1937) *Liebigs Ann.*, **531**, 1.

WEIGERT, F. AND MOTTRAM, J. C.—(1943) *Biochem. J.*, **37**, 497.—(1946) *Cancer Res.*, **6**, 97 and 109.

YOUNG, L.—(1950) Biochemical Society Symposia No. 5, p. 27.